# Functional interaction of human Ssu72 with RNA polymerase II complexes

**DOI:** 10.1371/journal.pone.0213598

**Published:** 2019-03-22

**Authors:** Benjamin M. Spector, Michael E. Turek, David H. Price

**Affiliations:** Department of Biochemistry, University of Iowa, Iowa City, Iowa, United states of America; Southern Illinois University School of Medicine, UNITED STATES

## Abstract

Phosphorylation of the C-terminal domain (CTD) of the large subunit of human RNA polymerase II (Pol II) is regulated during the transcription cycle by the combined action of specific kinases and phosphatases. Pol II enters into the preinitiation complex (PIC) unphosphorylated, but is quickly phosphorylated by Cdk7 during initiation. How phosphatases alter the pattern and extent of CTD phosphorylation at this early stage of transcription is not clear. We previously demonstrated the functional association of an early-acting, magnesium-independent phosphatase with early elongation complexes. Here we show that Ssu72 is responsible for that activity. We found that the phosphatase enters the transcription cycle during the formation of PICs and that Ssu72 is physically associated with very early elongation complexes. The association of Ssu72 with elongation complexes was stable to extensive washing with up to 200 mM KCl. Interestingly, Ssu72 ceased to function on complexes that contained RNA longer than 28 nt. However, when PICs were washed before initiation, the strict cutoff at 28 nt was lost. This suggests that factor(s) are important for the specific regulation of Ssu72 function during the transition between initiation and pausing. Overall, our results demonstrate when Ssu72 can act on early transcription complexes and suggest that Ssu72 may also function in the PIC prior to initiation.

## Introduction

Pol II transcription is a tightly regulated process that is, in part, controlled by the phosphorylation status of the CTD of the largest Pol II subunit. The human CTD is composed of 52 repeats of the consensus heptad sequence Y_1_S_2_P_3_T_4_S_5_P_6_S_7_ and each hydroxyl containing residue is specifically phosphorylated at certain stages of transcription by particular CTD kinases. The pattern of CTD phosphorylation generated by these kinases is further altered by CTD phosphatases to create a dynamic signal that recruits factors needed at different stages of transcription and RNA processing [1–3].

The transcription cycle begins with the formation of PICs on promoters and is followed by an orchestrated exchange of factors at each subsequent stage [1–3]. The first major transition occurs upon initiation during which the general initiation factors are replaced with pausing factors leading to the transient accumulation of paused complexes containing nascent transcripts on average 40–50 nucleotides long [4]. Pausing is due, in part, to the association of the DRB sensitivity inducing factor (DSIF) and the negative elongation factor (NELF) [5]. Although some of the paused complexes are converted into productive elongation complexes, most terminate rapidly [6]. The transition into productive elongation requires phosphorylation of DSIF by P-TEFb and this leads to the loss of NELF and the incorporation of the PAF1 complex [7]. Productive elongation continues until Pol II passes the polyadenylation signal at the 3′ end of genes and termination allows recycling of the polymerase [8].

CTD phosphorylation is dynamic during the transcription cycle. Pol II must be completely dephosphorylated prior to the formation of PICs [9]. Since Pol II is massively phosphorylated during transcription, reuse of terminated Pol II for new initiation must involve phosphatases. During initiation Ser5 and Ser7 are heavily phosphorylated by Cdk7, a subunit of the initiation factor TFIIH [10]. This phosphorylation is necessary for efficient capping and for proper pausing to take place [11]. Paused complexes that transition into productive elongation accumulate a dramatic increase in Ser2P at the 3′ end of genes [12]. Changes in Ser5P levels after initiation are less well defined and may be different in yeast and mammals [13–16]. Studies done primarily in yeast have provided evidence that Rtr1, the yeast homolog of mammalian RPAP2, and Ssu72 affect Ser5P on engaged Pol II [17–24]. Another phosphatase, Scp1, has also been reported to target Ser5P, but likely only effects the expression of specific genes rather than global transcription [25, 26]. Ser2P and Thr4P peak at the 3′ end of genes, aid in the recruitment of termination factors, and are then removed by FCP1. [14, 22, 27–30]. Tyr1P follows a similar pattern as Ser2P in yeasts, and is removed by Glc7 prior to termination [13, 31–33]. However, in mammals, Tyr1P is primarily found at promoters and enhancers with lesser signal at 3′ ends of genes [13].

When, where, and how phosphorylation marks on Ser5 are removed from engaged human Pol II is not completely understood. In part, this is due to many studies not differentiating between changes in Ser5P due to changes in the level of engaged Pol II or genuine changes in Ser5P phosphorylation on engaged Pol II. Results from yeast ChIP-chip experiments support a partial loss of Ser5P during transcription of the first few hundred base pairs [15, 16, 22]. However, mammalian ChIP-seq results suggest that the Ser5P signal does not decrease until the Pol II passes the polyadenylation signal at the 3′ end of genes [12–14]. It is not clear if these species differences are due to experimental limitations or actual mechanistic differences. RPAP2 and Ssu72 have been detected in the 5′ end of human genes by ChIP experiments [24, 34, 35], but it is not clear if they are active in PICs, during initiation, or on paused Pol II. The known interaction of yeast Ssu72 with initiation factors provides reasonable suspicion for activity prior to or during initiation, but this activity has never been directly studied [36–38]. Ssu72 is also an important factor involved in 3′-end formation and termination as a member of the CPF complex in yeasts and the CPSF complex in mammals [38, 39]. Given this, it would seem that Ssu72’s phosphatase function would occur only near the ends of genes, however, other works detailing Ssu72 mediated promoter-terminator interactions leave open possibilities of effects near initiation [40, 41]. Determining if human Ssu72 has phosphatase activity for early Ser5P removal and separating its potential activity from RPAP2 are essential steps towards understanding transcription.

Our prior detection of an early acting and metal ion-independent CTD phosphatase allowed us the opportunity to investigate the identity of the phosphatases involved in early dephosphorylation. Of the above phosphatases, RPAP2 and Ssu72 were the suspected phosphatases as Glc7, FCP1, and SCP1 are dependent on metal ions for function [42–44]. Here, we identify the early acting phosphatase as Ssu72, characterize its association on early transcription complexes, and determine characteristics of complexes susceptible to its activity.

## Results

### Early CTD phosphatase activity is magnesium-independent, salt-sensitive, and correlates with Ssu72

**Fig 1:**
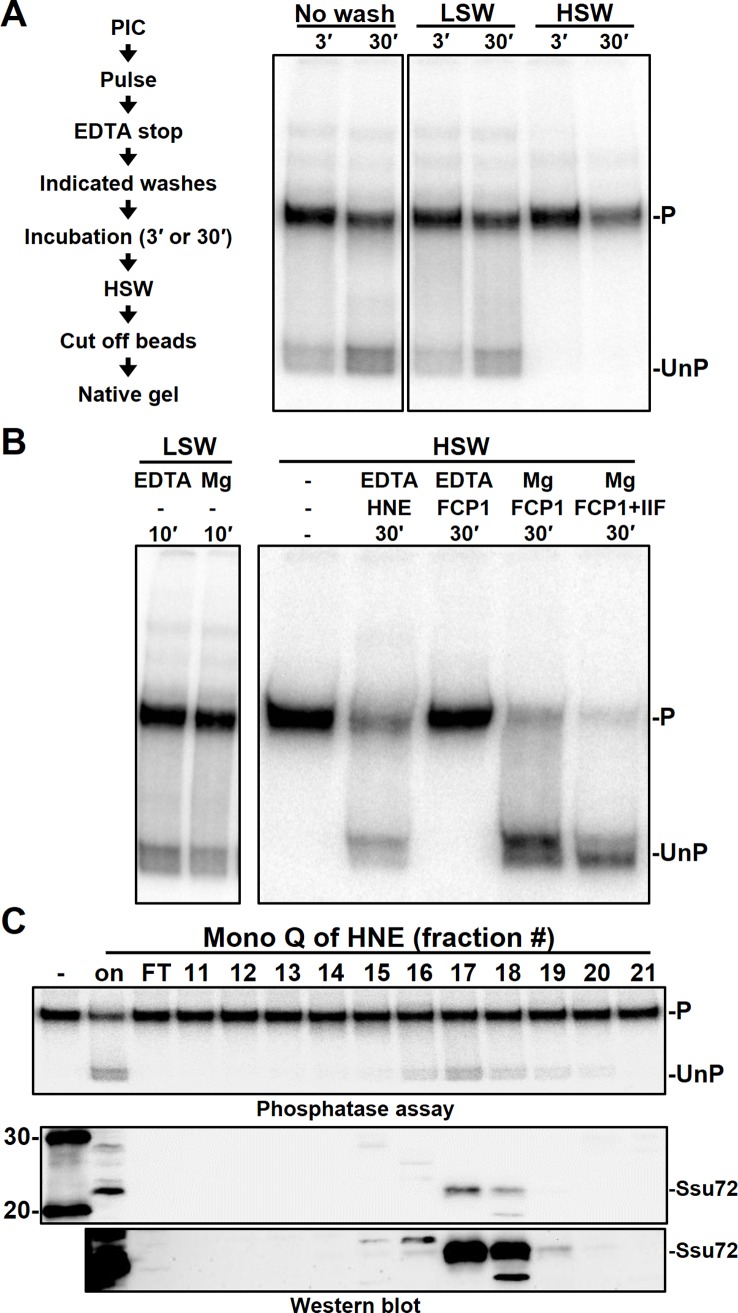
Characterization of the phosphatase activity acting on EECs. *A*, diagram of steps performed to analyze phosphatase activity (left) and EC-EMSA of EECs incubated for 3 or 30 minutes after stopping the pulse with EDTA followed by the indicated washes. LSW and HSW are low (60 mM KCl) or high (1.6 M KCl) salt washes. Details are in Materials and Methods. *B*, the effect of magnesium on phosphatase activity (left) and the contribution of FCP1 (right). EECs were washed as indicated and incubated with EDTA, Mg, HeLa nuclear extract (HNE), FCP1, or TFIIF (IIF) for the indicated times. Phosphorylated EECs (P); unphosphorylated EECs (UnP). *C*, Indicated Mono Q fractions of HNE were incubated for 30 minutes with high salt washed EECs that were resuspended in low salt wash (Phosphatase assay). The same fractions were blotted with antibodies to Ssu72 and shown at two different exposures (Western blot).

Previously we found using a human in vitro transcription system, that early elongation complexes are associated with an unknown phosphatase through the utilization of an elongation complex electrophoretic mobility shift assay (EC-EMSA) [11]. Before the identity of the phosphatase was investigated, these prior results that loosely characterized its association with the EC-EMSA were recapitulated. As described in the diagram (Fig 1A, left) this assay utilized a CMV-promoter-containing template immobilized on paramagnetic beads that was incubated with HeLa nuclear extract (HNE) for 30 minutes to form PICs. The complexes were initiated with a 30-second pulse containing 500 μM ATP/GTP, 1 μM UTP, and [α-^32^P]CTP (limiting UC pulse). Transcription was stopped with EDTA and the elongation complexes were then differentially treated with wash solutions. The wash conditions included a low (60 mM KCl) salt wash, a high (1.6 M KCl) salt wash, or no washing at all. Complexes were then incubated for an additional 3 or 30 minutes. After incubation, a final high salt wash (HSW) was done on complexes to remove all factors such that any mobility shift is due solely to CTD phosphorylation status. Complexes were then released from the beads with a 15-minute digestion with a restriction enzyme cutting upstream of the transcription-start-site (TSS) and analyzed on a native gel (Fig 1A). As previously reported, the mobility of the complexes was influenced by time of incubation and by the wash conditions prior to the incubation [11]. Complexes that were either unwashed or given a low salt wash (LSW) prior to incubation showed a shift to a higher mobility complex that increased in intensity with incubation time, whereas complexes initially given a HSW showed no such change. We have previously shown that this mobility change is due to changes in the phosphorylation status of the CTD as subsequent kinase reactions with P-TEFb restored high mobility complexes to low mobility complexes [11]. We conclude that a salt-sensitive and magnesium-independent phosphatase is acting on these complexes prior to high salt wash isolation.

To further confirm that phosphatase activity was causing the mobility shifts seen in the EC-EMSA, FCP1 and its activator, TFIIF, were utilized. Complexes were given either a LSW or a HSW after stopping the pulse with EDTA, resuspended in LSW buffer, and incubated with various combinations of TFIIF, FCP1, HeLa nuclear extract (HNE), EDTA, and magnesium for indicated amounts of time (Fig 1B). As expected, FCP1 with magnesium addbacks rescued the mobility shifts on complexes that were given a HSW and this effect was blocked by EDTA (Fig 1B). This FCP1 and magnesium rescue effect was stimulated by TFIIF. Thus, the mobility shift is further confirmed to be due to the action of a CTD phosphatase and FCP1 is not the phosphatase that is working on EDTA stopped complexes. Notably, complexes that had only magnesium added back after a LSW showed no increase in phosphatase activity when compared to complexes that had only had EDTA added back. Since the LSW only removes unbound or loosely associated factors, only magnesium-independent phosphatases are directly associated with early elongation complexes (EECs). Finally, the phosphatase can be added back once washed away as HNE addbacks to HSW isolated complexes rescued phosphatase activity (Fig 1B).

To aid in the identification of the phosphatase, chromatography of HNE on Mono Q was performed and each fraction was examined for phosphatase activity when added back to HSW isolated EECs in the presence of EDTA. After incubating isolated elongation complexes with each fraction for 30 minutes, complexes were then assayed for the mobility shift caused by phosphatase activity (Fig 1C, top). Fractions 16 through 20 yielded phosphatase activity on EECs with fractions 17 and 18 showing the most activity. All the fractionations were then western blotted for Ssu72 (Fig 1C, bottom). Each fraction that contained phosphatase activity yielded specific bands for Ssu72 at 24 kDa, but to differing amounts. Fractions 17 and 18 both contained large amounts of Ssu72 in comparison to the other fractions. Surprisingly, the amount Ssu72 activity did not linearly follow the amount of Ssu72 added back. It is possible that the phosphatase assay is not linear due to differences in specific phosphorylation patterns in individual molecules within the population of EECs. Some patterns may be easily phosphatased and other patterns may require more Ssu72. Alternatively, the non-linearity could be caused by a lack of co-fractionation of additional factors involved in facilitating add-back of Ssu72. Overall, these results strongly implicate Ssu72 as the early-acting phosphatase.

### Ssu72 is associated with early transcription complexes

**Fig 2:**
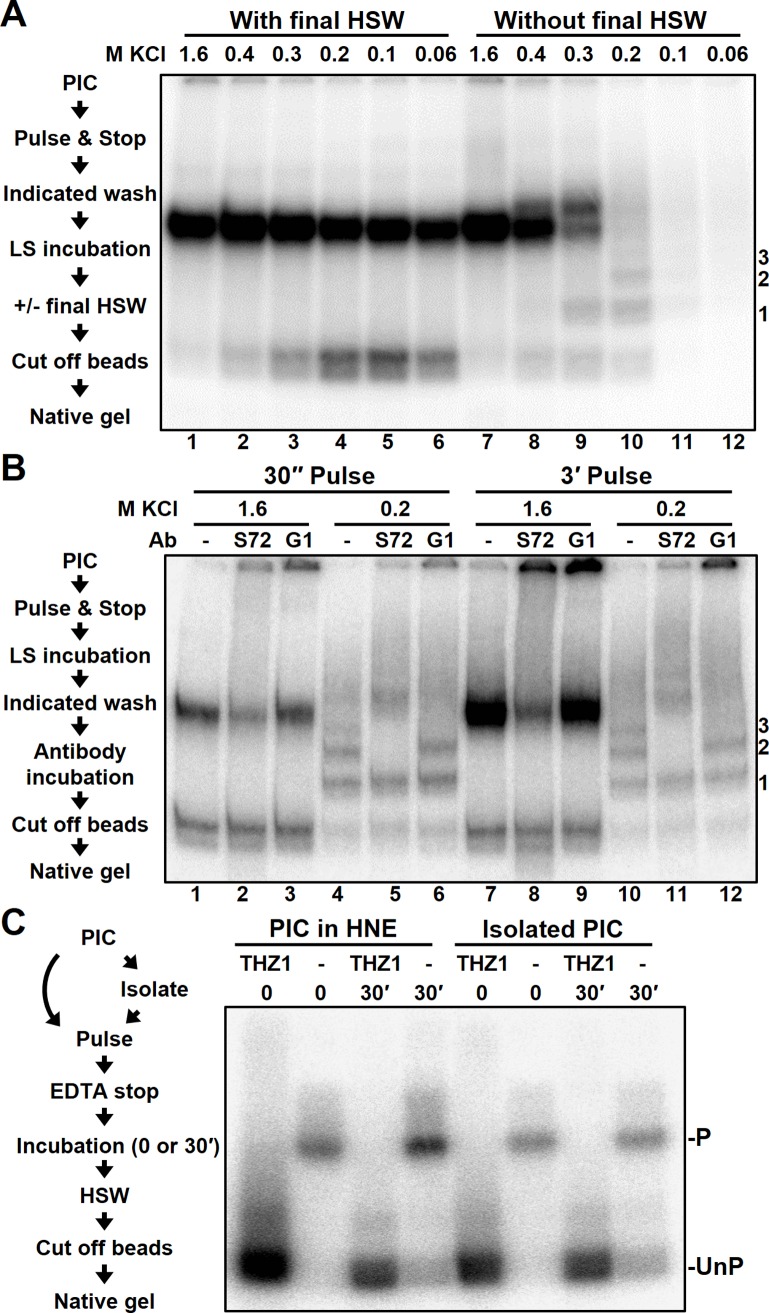
Association of the phosphatase activity with EECs & PICs. *A*, diagram of steps performed to analyze salt-sensitivity of the phosphatase activity (left) and EC-EMSA of EECs incubated for 30 minutes after stopping a limiting UC pulse with EDTA, washing with the indicated KCl concentration washes, and washing first replicate with one final HSW prior to restriction (right). LSW and HSW are low (60 mM KCl) or high (1.6 M KCl) salt washes. Details are in Materials and Methods. Phosphatased bands with altered mobility are labeled 1, 2 and 3. *B*, diagram of steps performed to identify components of intermediate complexes (left) and EC-EMSA of complexes that were pulsed as indicated, stopped with EDTA, washed as indicated, and blotted with Ssu72 antibody (S72) and Gdown1 (G1) antibody after a 30’ low salt incubation. *C*, Diagram of steps performed to determine if the phosphatase associates with PICs (right) and EC-EMSA of EECs generated from PICs with and without THZ1 and HNE and incubated for indicated times.

To further examine the stability of the phosphatase interaction with Pol II elongation complexes, limiting UC-pulsed and EDTA-stopped complexes were immediately washed with buffers containing decreasing amounts of salt. After this initial wash, the complexes were incubated in EDTA containing LSW buffer for 30 minutes to allow phosphatase activity. Complexes were then either given a final HSW prior to being released from the beads by restriction digestion or they were released without a final HSW (Fig 2A, diagram on left). Complexes that received a salt wash of greater than 200 mM prior to incubation lost a significant amount of phosphatase activity as seen after a final HSW (Fig 2A, lanes 1–3). Without a final HSW the recovery of elongation complexes was reduced by salt concentrations less than 200 mM (Fig 2A, lanes 11–12). Evidently, salt washes of greater than 400 mM were necessary for the restriction enzyme to efficiently remove complexes from the beads and this was especially true for phosphorylated complexes (Fig 2A, lanes 7–8). The phosphatased complexes seen after 200 or 300 mM salt wash were not uniformly of one mobility (lanes 9–10), but instead provided evidence of associated factors causing reduced mobility (Fig 2A, complexes 1–3).

To determine if one of the lower mobility elongation complexes might be due to association of Ssu72 and to see if a longer pulse would affect the results, either 30-second or 3-minute limiting UC-pulsed complexes were washed with 200 mM salt and then incubated with antibodies to either Ssu72 or Gdown1, a substoichiometric subunit of Pol II (Fig 2B, diagram on left). Of the complexes that were released in either pulse condition, two of the lower mobility complexes identified above (complexes 2 and 3) were supershifted by the Ssu72 antibody (Fig 2B, lanes 5 and 11) confirming that Ssu72 can associate with EECs. However, this association is not completely stable since some of the complexes dephosphorylated by Ssu72 did not retain the phosphatase. In addition, Ssu72 antibodies produced a lower mobility smear above the phosphorylated complexes suggesting that phosphorylated complexes may also contain Ssu72. We probed for Gdown1 because complex 1 resembled the shift caused by that protein [45, 46]. However, Gdown1 antibodies had only a minor effect on the lowest mobility complex (complex 3). It is unclear if Ssu72 requires a protein from these differentially mobile complexes to associate or if it may associate with the Pol II elongation complex independently. Interestingly, a smaller fraction of the 3-minute pulse complexes was phosphatased (lanes 7–9). The reason for this will be addressed below.

To determine if the phosphatase enters the transcription complex before or after initiation, we examined if it could associate with PICs. PICs were formed on the immobilized DNA template and, prior to pulsing, were repeatedly washed in low salt buffer (Fig 2C, diagram on left). This process removes soluble and loosely associated proteins and prevents their activity in transcription. THZ1, a Cdk7 inhibitor, was utilized during PIC formation to provide maximally unphosphorylated controls. Significant phosphatase activity was found regardless of the removal of unbound or loosely bound proteins (Fig 2C). This result indicates that the phosphatase joins transcription complexes during preinitiation complex formation.

### Ssu72 activity is affected by P-TEFb function

**Fig 3:**
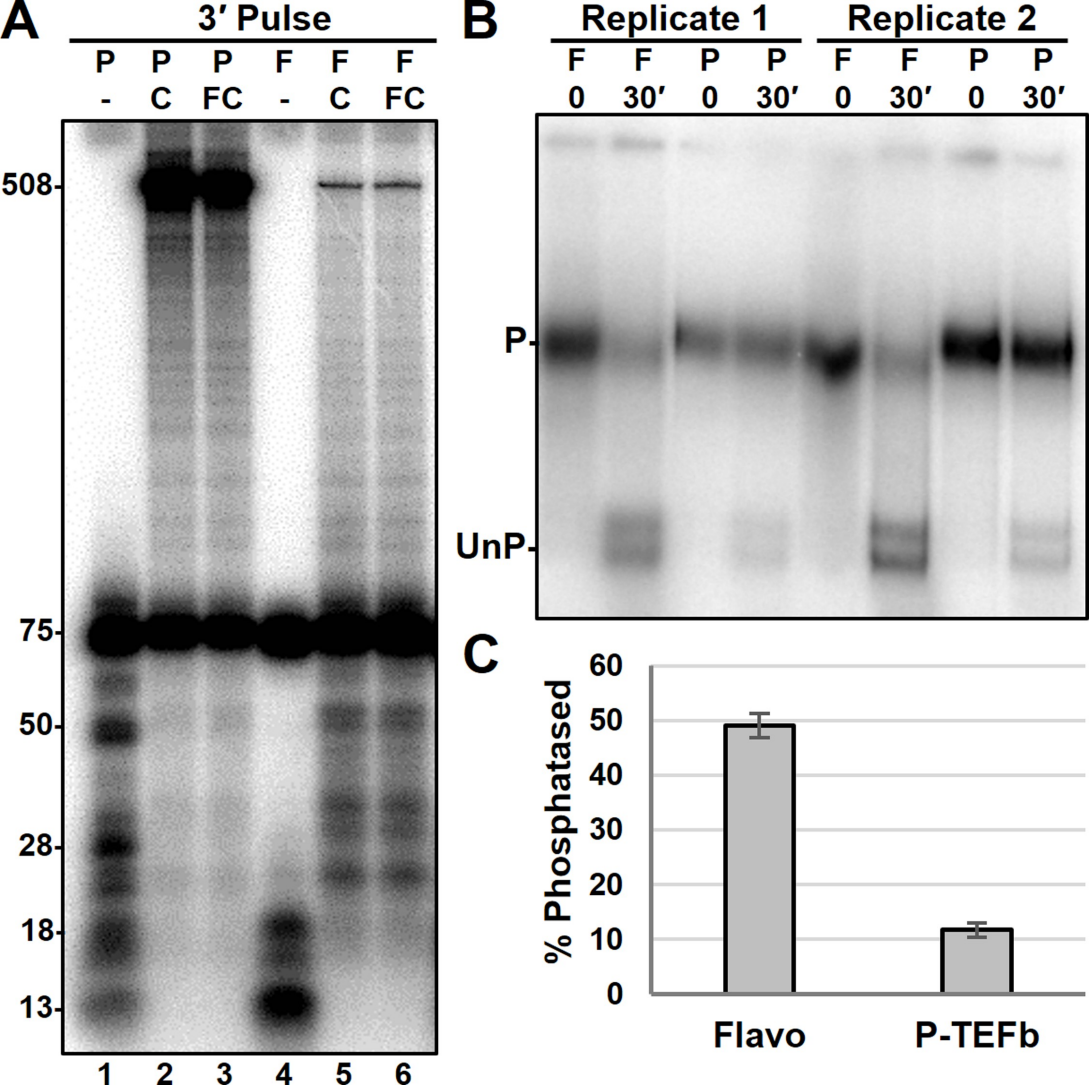
Influence of P-TEFb on the phosphatase assay. *A*, RNA generated during a 3-minute limiting UC pulse with or without a subsequent chase on a template with a 503 nt runoff. Excess P-TEFb (P) or flavopiridol (F) were added during PIC formation. Indicated complexes were chased (C) for 3 minutes in the absence or presence of flavopiridol (FC). *B*, EC-EMSA of EECs generated in the presence of flavopiridol or P-TEFb and pulsed with limiting UC for 3 minutes. Complexes were stopped with EDTA and incubated for the indicated times prior to HSW (1.6 M KCL) and LSW (60 mM KCl). *C*, quantification of phosphatase activity in *B*. Total amount of counts within the unphosphorylated band (UnP) was divided be total counts within the lane to determine percent phosphatased.

Knowing that Ssu72 is present in EECs we wondered if it would remain associated during the next step of transcription–the transition into productive elongation driven by P-TEFb. PICs were formed in the presence of either the P-TEFb inhibitor, flavopiridol, or in the presence of excess P-TEFb and were then pulsed with a 3-minute limiting UC pulse. This 3-minute pulse condition was used to ensure that P-TEFb had adequate time to function before the EDTA stop and incubation [47]. P-TEFb function was verified by chasing RNAs generated from the 3-minute pulse with or without flavopiridol in the chase (Fig 3A, lanes 2–3). Should P-TEFb function during the pulse, flavopiridol addition during the chase will not prevent complexes from reaching runoff as productive elongation complexes [47]. As expected, complexes treated with flavopiridol during PIC formation failed to enter productive elongation (lanes 4–6), whereas P-TEFb treated complexes were able to reach run-off with a flavopiridol containing chase indicating that P-TEFb acted during the long pulse.

**Fig 4:**
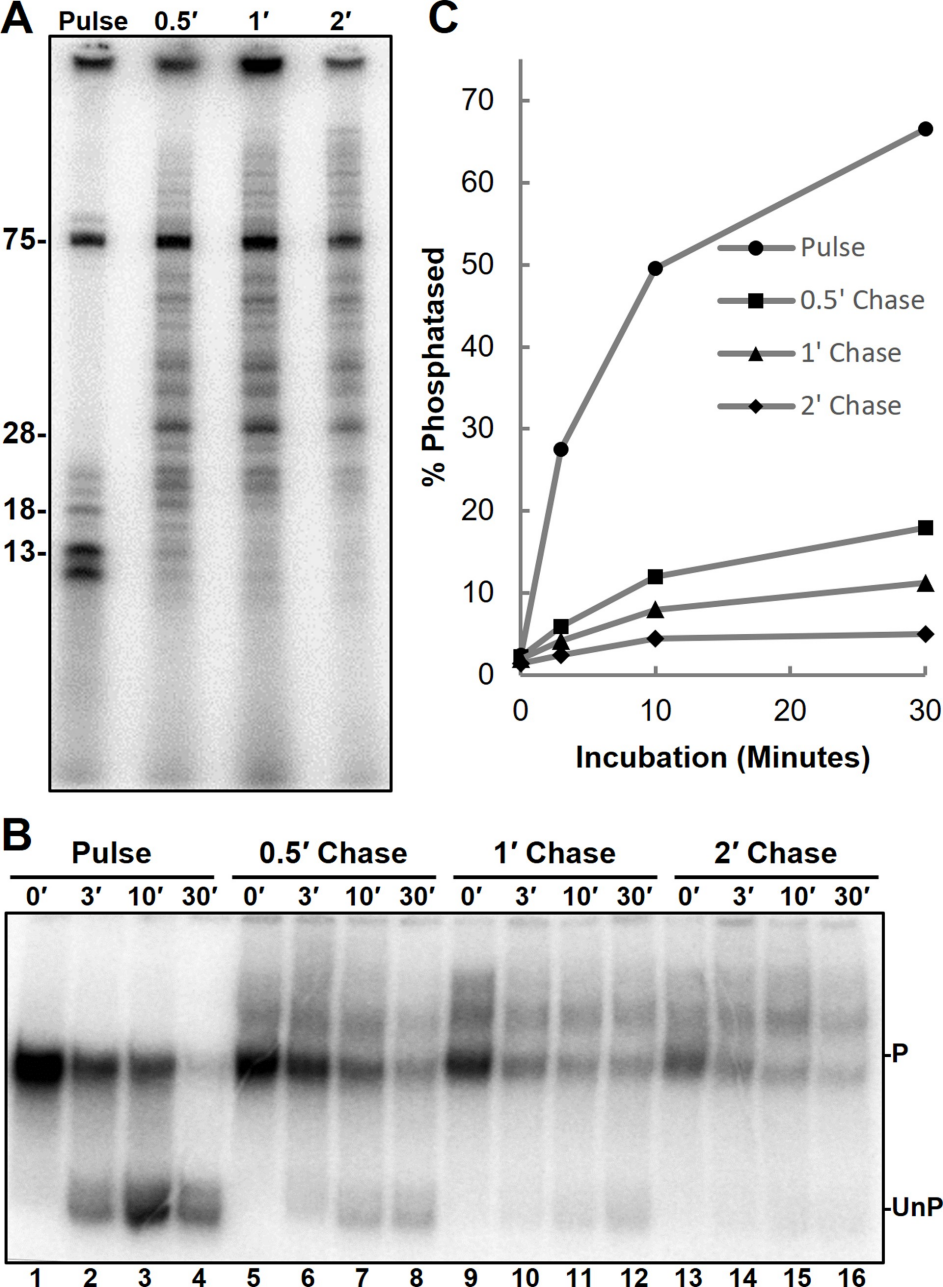
RNA length effect on phosphatase activity. *A*, RNA generated during a 30 second limiting UC pulse in the presence of flavopiridol were chased for 0, 0.5, 1, and 2 minutes. *B*, EC-EMSA of complexes containing transcripts like those in *A* were then incubated for indicated times. *C*, Quantification of relative phosphatase activity by dividing total UnP counts by total lane counts.

The long pulse condition was then utilized to investigate a P-TEFb effect on Ssu72 activity. To do so, PICs were formed with excess P-TEFb or with flavopiridol, pulsed, and stopped with EDTA. Complexes were then either incubated 30 minutes prior to HSW or immediately given a HSW to prevent Ssu72 activity (Fig 4B). In both replicates of this experiment, the presence of flavopiridol caused the amount of phosphatased complexes observed after the 30-minute incubation to be significantly increased compared to P-TEFb treated complexes, which suggests stronger and more complete Ssu72 activity (Fig 3B and 3C). However, P-TEFb could have altered the amount of observable Ssu72 activity through several different mechanisms. Firstly, excess P-TEFb could have caused an increase in Ser2 phosphorylation which might mask Ssu72 activity. Secondly, the effect could have been mediated by RNA length since the long pulse with P-TEFb leads to slightly longer transcripts. Many factors, such as DSIF, require RNA to be of an adequate length for proper binding and the binding of one such factor may displace or inhibit Ssu72 activity [48]. Other possible explanations include P-TEFb mediated loss of Ssu72 from the elongation complex or kinase mediated inhibition of Ssu72 activity. Overall, these results cannot determine how P-TEFb is affecting Ssu72 activity but do reveal that P-TEFb can affect the activity detected.

### Ssu72 activity depends on the length of the nascent RNA

To determine if the effect of P-TEFb on Ssu72 was due to an effect of transcript length, Ssu72 activity was measured on flavopiridol treated complexes under chase conditions that generated RNAs of different lengths (Fig 4A). The kinetics of Ssu72 activity on the complexes with different length RNA were followed using EC-EMSA with time points at 0, 3, 10, and 30 minutes. As expected the complexes containing short transcripts displayed high levels of phosphatase activity (Fig 4B, lanes 1–4). However, increasing the length of the RNA had a strong, length-dependent negative impact on Ssu72 function (Fig 4B and 4C, lanes 5–16). This indicates that Ssu72 activity is lost upon RNA extension.

**Fig 5:**
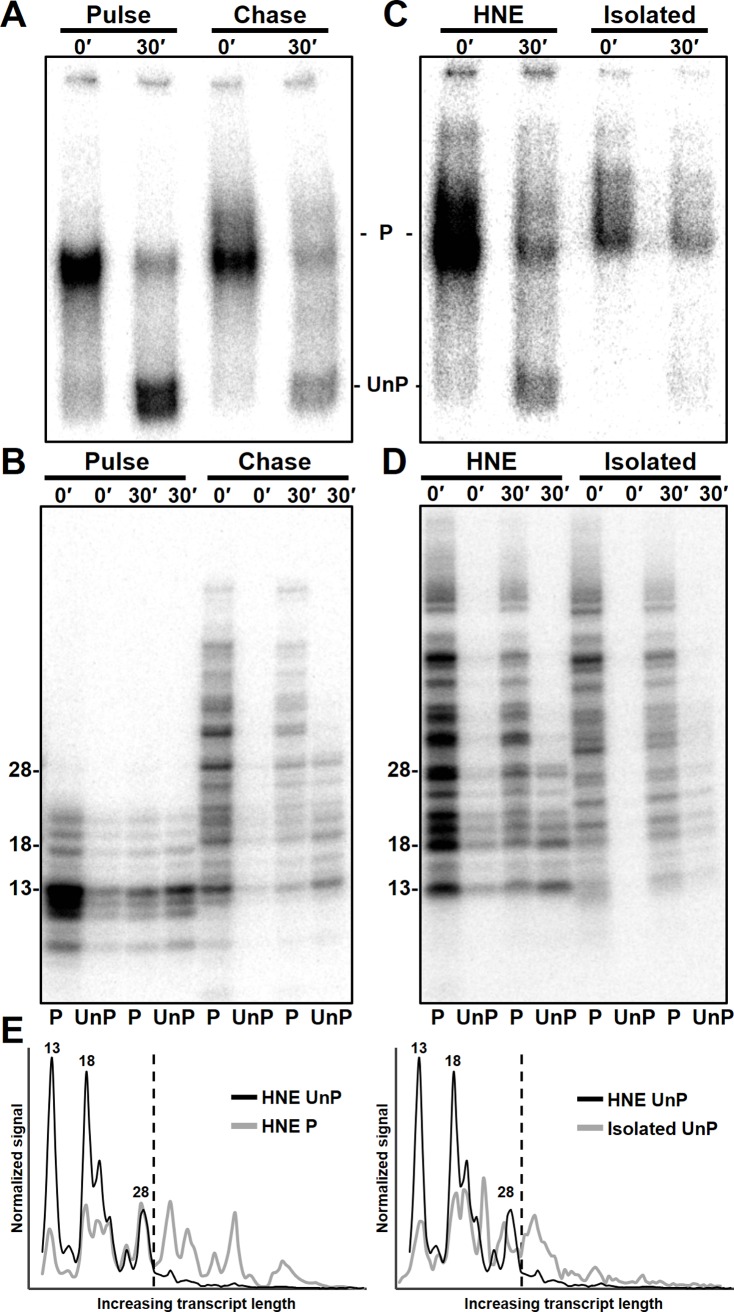
RNA length restriction for phosphatase activity and potential factor involvement. *A*, EC-EMSA of EECs generated from a 30-second limiting UC pulse with and without a 30-second chase. Complexes were incubated for 0 or 30 minutes prior to HSW isolation. *B*, RNAs associated with phosphorylated (P) and unphosphorylated (UnP) complexes were extracted from EC-EMSA gel in *A* and analyzed by urea PAGE. *C*, EC-EMSA of EECs generated from PICs in the presence of HNE or from PICs that were isolated before the pulse. Each were given a 30-second limiting UC pulse and a 30-second chase, stopped with EDTA, and either incubated for 0 or 30 minutes. *D*, RNAs associated with each EC-EMSA complex from *C* were analyzed as in *B*. *E*, The normalized profile of transcripts associated with the unphosphorylated complexes after 30 minutes of phosphatase function (HNE UnP) was compared to RNA from the phosphorylated complex generated during the pulse (HNE P) or to RNA associated with unphosphorylated complexes generated from isolated PICs that were incubated for 30 minutes (Isolated UnP). Specific transcript sizes are indicated and a dotted line demarcates the position of the loss of phosphatase activity in HNE.

To further investigate this RNA length-dependence on Ssu72 activity, early elongation complexes with both short and long transcripts were generated and subjected to EC-EMSA after allowing the phosphatase time to function. PICs were generated in the presence of flavopiridol, limiting UC-pulsed, and either immediately stopped or chased for 30 seconds and then stopped. These complexes were then either immediately isolated or given a 30-minute incubation prior to isolation and then separated via EC-EMSA. As expected the complexes that were not chased exhibited high phosphatase activity and those whose transcripts were extended during the chase displayed less phosphatase activity (Fig 5A). To determine if there were differences in the lengths of RNAs in the phosphorylated and unphosphorylated complexes, bands were excised and soaked out of the EC-EMSA. The RNA was then extracted from the supernatant of the gel solution and run in urea-PAGE. The amount of RNA isolated from the phosphorylated and unphosphorylated complexes from with and without Ssu72 function mirrored the amounts of the complexes seen in the EC-EMSA demonstrating that recovery of the RNA was good (Fig 5B, Pulse). Transcripts from the limiting UC pulse were all less than about 25 nt and there were no differences between the phosphorylated and unphosphorylated complexes (Fig 5B, Pulse). After a 30 second chase a wide range of transcript sizes were found (Fig 5B, Chase). We were surprised to find that all transcripts in the unphosphorylated complexes were less than 28 nt even though about half of chased transcripts were longer (Fig 5B, Chase 30′ UnP). These results demonstrate that that Ssu72 activity is lost after the transcript reaches 28 nt. Some of the short transcripts were present in the phosphorylated complexes suggesting that Ssu72 is limiting (Fig 5B, Chase 30′ P). The length-dependence was reproduced multiple times under different reaction conditions (S1 Fig). Additionally, we determined that the length dependence is not affected by cap status of the RNA because reactions carried out in the presence of hydrogen peroxide that blocks capping [11] still displayed the length dependence (S1 Fig).

Finally, we decided to address a likely mechanism for the RNA length-dependence on Ssu72 function involving loss of initiation factors and/or association of elongation factors with the elongation complex. Because the effects of elongation factors that associate after initiation can be eliminated via PIC washes, we compared transcripts in phosphorylated and unphosphorylated complexes generated in the presence of extract to those generated from isolated PICs. As found earlier the phosphatase activity was present under both conditions, but as compared to Fig 2C. the level of activity was less due to the fact that some of the RNAs were longer than 28 nt due to the use of a 30 second chase (Fig 5C, Isolated). However, phosphatase activity on isolated complexes was also diminished in comparison to similarly chased complexes generated in the presence of HNE, which suggests phosphatase losses due to the PIC washes (Fig 5C). These losses during the PIC isolation were not previously observed due the effects of P-TEFb on unwashed complexes since flavopiridol was not utilized (Fig 2C). Analysis of RNA in the excised bands from the EC-EMSA demonstrated again that EECs in the presence of HNE exhibited the 28 nt cutoff for phosphatase activity (Fig 5D, HNE 30′ UnP). However, there was an increase in length of transcripts found in the unphosphorylated complexes generated from washed PICs (Fig 5D, Isolated 30′ UnP). This effect can be best seen in normalized lane profiles of the RNA gel that highlight the enrichment of short transcripts in unphosphorylated complexes relative to phosphorylated complexes in the presence of HNE (Fig 5E, left) and that directly compare the lengths of transcripts associated with unphosphorylated complexes from both washed (Isolated) and unwashed (HNE) PIC conditions (Fig 5E, right). Therefore, it is likely that as EECs travel downstream in the presences of all factors, the phosphatase’s ability to act is rapidly lost due to the action of one or more factors.

## Discussion

**Fig 6:**
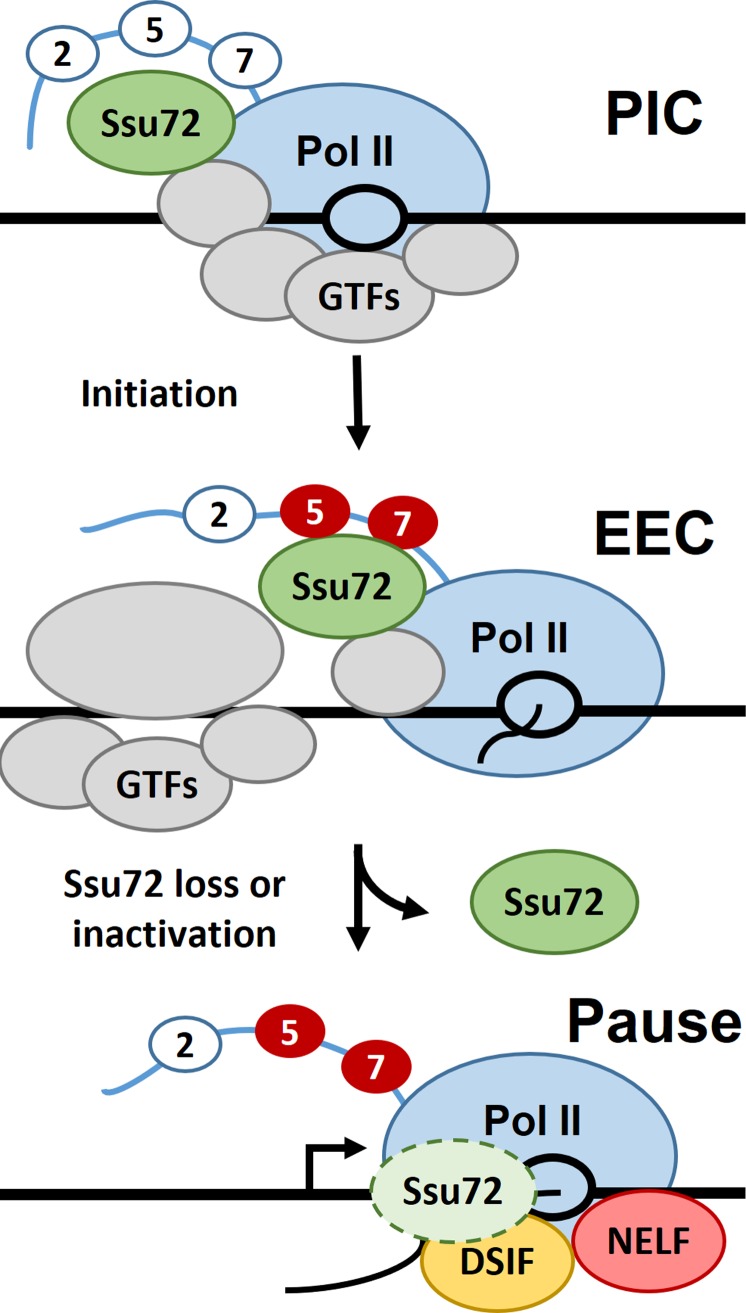
Summary of results concerning association of Ssu72 with transcription complexes. Transcription complexes at the three indicated stages are shown with sites of CTD phosphorylation (red) indicated. General transcription factors (GTFs) involved in initiation and pausing factors DSIF and NELF are shown. Our results indicate that Ssu72 is present in the PIC and early elongation complexes and is either lost or inactivated before the paused state.

We have examined association of Ssu72 with Pol II during early stages of transcription. Ssu72 becomes associated with Pol II during PIC formation, remains associated during very early elongation, and then is either inactivated or removed as complexes enter the paused state (Fig 6). We demonstrated that Ssu72 is capable of dephosphorylating early transcription complexes and uncovered a surprising transcript length-dependence for the phosphatase activity. Through unique usage of the EC-EMSA, we were able to remove the effects of other CTD phosphatases to specifically measure the rate and efficiency of Ssu72 activity. Initial experiments with this assay revealed that EECs only have EDTA-resistant phosphatase activity associated and that only addbacks containing Ssu72 could rescue this EDTA-resistant phosphatase activity. Phosphatase activity remained associated with EECs after washing with up to 200 mM KCl and antibody supershifts were used to correlate physical association of Ssu72 protein with phosphatase activity. Isolation of PICs prior to initiation did not eliminate the phosphatase activity on EECs. This indicates that the phosphatase becomes associated with the transcription complex during PIC assembly. Chasing EECs in the presence of extract resulted in the loss of phosphatase activity once transcripts exceeded 28 nt in length. Removal of unbound factors or factors loosely associated with the PIC abrogated the distinct transcript length restriction. Therefore, during the transition between initiation and pausing, the action of unknown factor(s) eliminates phosphatase activity on transcription complexes (Fig 6). Overall, we confirmed an earlier study showing that a CTD phosphatase was associated with EECs [11], identified it as Ssu72, demonstrated association of phosphatase activity with PICs, and discovered that Ssu72 activity was lost as nascent transcripts were extended beyond 28 nt.

Our studies have refined the role of Ssu72 during transcription and distinguished it from other CTD phosphatases. We did not detect any magnesium-dependent phosphatase activity associated with early elongation complexes demonstrating that the magnesium-dependent phosphatases, FCP1, Scp1, and Glc7 do not function during that time. FCP1 is likely the key phosphatase associated with free Pol II to protect it from phosphorylation prior to PIC formation [29, 30], Scp1 only effects the expression of specific genes [26], and Glc7 is primarily the Tyr1 phosphatase that functions at the 3′ end during termination [32]. Determining that Ssu72, not RPAP2, was responsible for the observed phosphatase activity was more challenging since two studies on RPAP2 (Rtr1 in yeast) arrived at conflicting conclusions regarding its inhibition by EDTA [19, 49]. However, it is known that RPAP2 relies on scaffold proteins that preferentially bind a phosphorylated CTD [50, 51]. Therefore, washing the PICs prior to initiation should result in significant loss of these scaffold proteins which would prevent retention and activity of RPAP2 on subsequently generated EECs. The similar level of phosphatase activity after PIC isolation and the loss of phosphatase activity following the loss of Ssu72 association would then seem to indicate that RPAP2 plays little to no role in dephosphorylating very early human transcription complexes.

It is clear that Ssu72 can function in early elongation, but the effects of this activity are subjects of further study. Ssu72 phosphatase activity could be important for modulating the phosphorylation state of the CTD to possibly set the stage for later factor exchanges, but it is also possible that Ssu72 serves some function unrelated to its phosphatase activity as suggested previously in yeast [17]. It is also possible that Ssu72 plays a role in abnormally early stopping of Pol II caused by backtracking or DNA damage before the pausing factors are incorporated. These possible functions of Ssu72 may also require another protein as a scaffold or bridge to allow Ssu72 to associate with transcription complexes since another protein was evident on 200 and 300 mM salt washed EECs. The identity and potential role of this protein was not determined. However, it is clear that if there is a Ssu72 bridging factor, it can interact with EECs that have been phosphatased. Given our results, we propose a model for Ssu72 function that places Ssu72 within PICs and EECs prior to pausing (Fig 6). As transcripts elongate past 28 nt, Ssu72 is either lost from the elongation complex or is inactivated due to some exchange of factors prior to pausing. If Ssu72 remains associated but inactivated, it could be reactivated by removal of the inhibitor at the 3′ end of genes.

Ssu72 association with PICs utilizing a short runoff template suggests that Ssu72 may affect initiation independent of its 3′ end functions. Ssu72 mediated effects on initiation have previously been hypothesized partially due to its roles in promoter-terminator gene looping and 3′ end phosphatase activity, but very few studies have examined its effects independent of these roles [40, 52]. The few studies that have examined effects of Ssu72 in an in vitro transcription system have shown defects in elongation following Ssu72 loss, but only suggested possible initiation effects [21, 53]. Since our assay eliminates 3′ end effects and reveals a PIC associated phosphatase, we propose that Ssu72 affects some aspect of PIC formation or stability. Although we do not know how Ssu72 would affect these processes, previous studies on PIC formation and stability reveal a few possibilities. Firstly, it has been shown that TFIIB has rapid turnover within PICs prior to Pol II association [54]. Since Ssu72 seemingly allows for TFIIB to adopt the open conformation needed for DNA binding, Ssu72 may facilitate PIC formation via effects on TFIIB [55]. Secondly, prior work shows that Cdk7 can partially function prior to initiation of transcription and that inhibition of Cdk7 leads to increased half-lives of template bound initiation factors [56]. As such, Ssu72 function within PICs may oppose Cdk7 activity to stabilize PICs in a similar manner. Finally, Ssu72 may function to remove trace CTD phosphorylations on Pol II attempting to join the PIC, which may then serve to enhance the association of Mediator due to its inability to associate when the CTD is phosphorylated [57]. Mediator and Ssu72 are both important for the proper compaction of the yeast genome and both proteins co-precipitate from chromatin protein extracts [58, 59]. This leads to the possibility that Ssu72 effects on chromatin are a result of its roles with Mediator and the PIC.

Until now, specifics of Ser5P removal during human transcription were unclear due to the redundant roles of many CTD phosphatases and potential differences from yeast in which most studies have been carried out. Because yeast do not have promoter proximal pausing, factors associated with early elongation complexes are likely different. We do not know if the yeast Rtr1 homolog RPAP2 plays a role at specific human genes, but our in vitro data suggests that in humans, Ssu72 is the primary phosphatase associated on very early elongation complexes. RPAP2 could become associated after pausing factors are released. Our findings lead to intriguing questions regarding the role that Ssu72 plays at early stages of human transcription and how it sets the stage for further CTD modifications. Future investigations into the role of the Pol II CTD during the transcription cycle will need to account for the effects of Ssu72 activity early in transcription and how it may affect PIC assembly, initiation, and later exchanges of factors.

## Materials and methods

### HNE fractionation

A total of 0.5 ml of HNE containing 300 mM KCl was diluted four times to a final salt concentration of 75 mM with HGEDP (25 mM HEPES, pH 7.6, 15% glycerol, 0.1 mM EDTA, 1 mM DTT, and 0.1% PMSF). After sitting on ice for 10 minutes the protein sample was centrifuged at 17,000 g for 20 minutes at 4°C. The supernatant was loaded onto a 1-mL Mono Q column that was pre-equilibrated with 75 mM HGKEDP and flow-through fractions were collected. The column was washed with 5 column volumes of 75 mM HGKEDP followed by elution with a linear gradient from 75 to 500 mM HGKEDP and a step to 1M. All fractions were stored at -80°C. Fractions were run in a 10% SDS-PAGE gel and western blotted utilizing the Ssu72 antibody (N1C3) at 1:90000.

### In vitro transcription assay

Production of nascent transcripts in early elongation complexes has been previously described [48]. Typical reactions contained 1 μl HNE in a total reaction volume of between 8 and 14 μl. Preinitiation complexes (PIC) were formed by incubating immobilized CMV promoter containing DNA templates -800 to +175 or -800 to +508 with HNE for 30 minutes in the presence of 60 mM KCl, 5 mM MgCl_2_, 20 mM HEPES (pH 7.6), 1 mM DTT, and 0.5 U/μl SUPERase-In. Where indicated, PICs were also formed in the presence of 1 μM flavopiridol, 1 μM THZ1, or excess P-TEFb [60]. Initiation was accomplished with 3-minute or a 30-second pulses containing 500 μM ATP/GTP/UTP with limiting [α-^32^P]CTP or containing 500 μM ATP/GTP with limiting UTP at 1 μM and limiting [α-^32^P]CTP. If chased, the concentration of all nucleotides were brought to 500 μM. If complexes were to be isolated, see EC-EMSA protocol. If RNA was to be isolated, elongation complexes were stopped with stop solution containing 100 mM Tris pH 7.6, 0.2 mg/ml Torula Yeast RNA, 20 mM EDTA, and 1% Sarkosyl (Sigma, R6625). The RNA was then isolated by phenol extraction and precipitated by addition of 3 volumes of 95% ethanol containing 0.5 M ammonium acetate. The resulting pellet was then washed with 70% ethanol and resuspended in RNA loading buffer (9.35 M Urea, 0.25x TBE, 0.0075% bromophenol blue, and 0.03% xylene cyanol). Samples were then run on urea polyacrylamide denaturing gels (6 M urea). Gels were then dried, exposed to an imaging plate, and visualized by phosphorimaging (Fuji FLA-7000).

### EC-EMSA

EC-EMSAs were described previously [48]. Elongation complexes were formed as described above, but instead were stopped with EDTA in various salt conditions (60 mM or 1.6M KCl). These complexes were then incubated for indicated times, typically 30 minutes, and were subjected to a final high salt wash of 1.6 M KCl unless otherwise indicated and resuspended in low salt. Any addbacks took place immediately after LSW resuspension and prior to incubation. Complexes were then incubated with the restriction enzyme SacI for 15 minutes to free them from the paramagnetic beads by cutting 17 base pairs upstream of the TSS. After digestion was halted via addition of EDTA containing loading buffer, complexes were then incubated with indicated antibodies for 15 minutes prior to loading and electrophoresis. Complexes were run on 4% acrylamide (37.5:1 acrylamide:bis) gel in a 0.5 x Tris/glycine buffer for 2 hours at 6 watts at room temperature. Resulting gels were then dried and visualized by phosphorimaging (Fuji FLA-7000). In Fig 5B and 5D, elongation complexes from the native gel were cut out and soaked overnight with a buffer containing 20 mM Tris, 1 mM EDTA, 250 mM KCL, and 1% SDS. The RNA from these complexes were then phenol extracted, ethanol precipitated, and separated on denaturing RNA gels utilizing the previous method.

## Supporting information

S1 Fig Effect of capping on the RNA length-dependence of the phosphatase. RNA extracted from phosphorylated (P) and unphosphorylated (UnP) EECs generated from 30 or 60 second limiting C pulses and 30 minute incubations. In one of the repeated experiments 0.3 mM peroxide was added during initiation to inhibit capping.
(TIF)
