# Supplementary figures and images for: Functional interaction of human Ssu72 with RNA polymerase II complexes
Source: PLoS One. 2019 Mar 22;14(3):e0213598. doi: 10.1371/journal.pone.0213598 (PMC6430399; doi:10.1371/journal.pone.0213598)

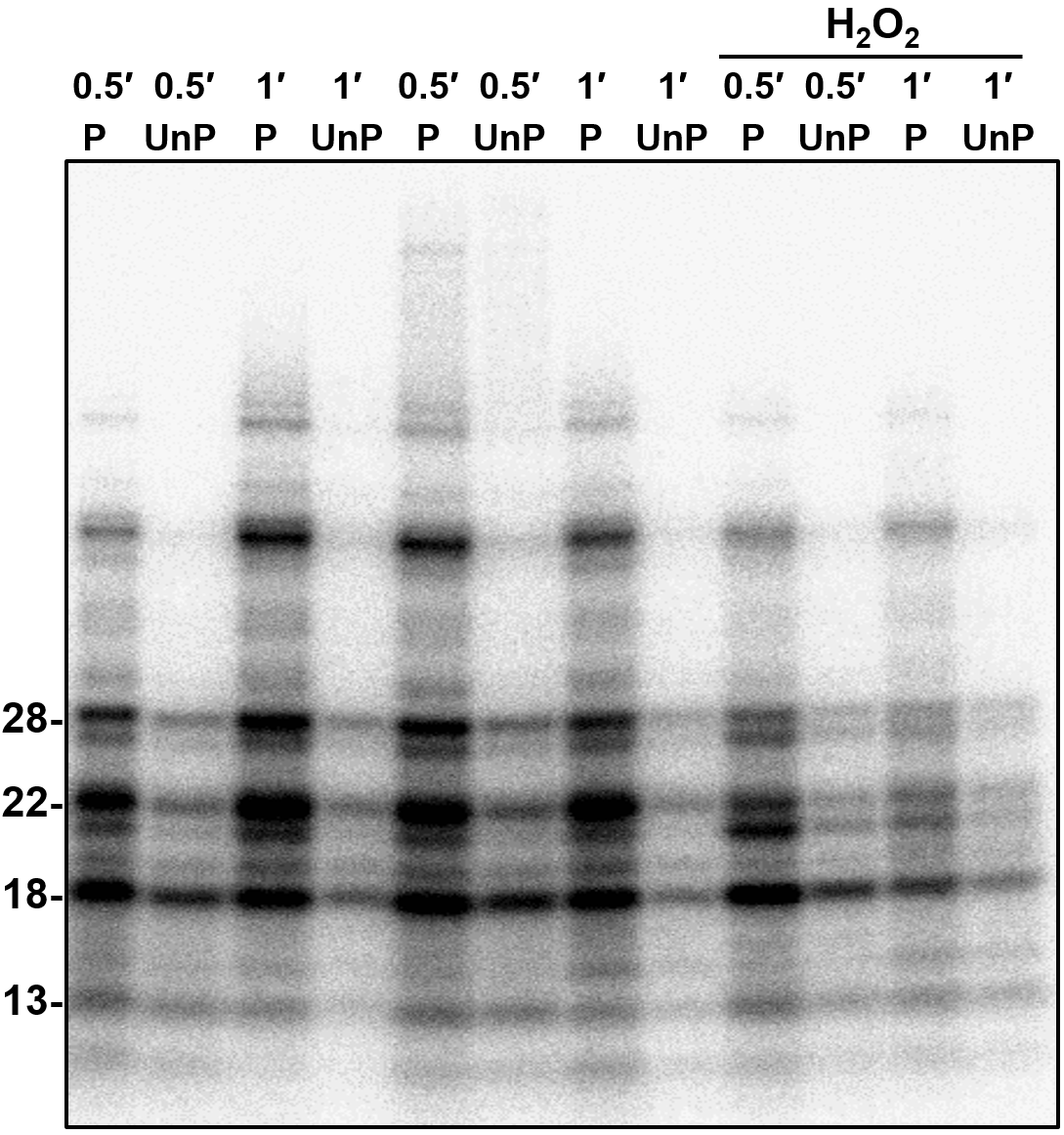

Supplement: S1 Fig — RNA extracted from phosphorylated (P) and unphosphorylated (UnP) EECs generated from 30 or 60 second limiting C pulses and 30 minute incubations. In one of the repeated experiments 0.3 mM peroxide was added during initiation to inhibit capping. (TIF) [file pone.0213598.s001.tif]
